# Hand Colonization with Gram-Negative Organisms of Healthcare Workers Accessing the Cardiac Intensive Care Unit: A Cross-Sectional Study at the Uganda Heart Institute

**DOI:** 10.1155/2019/6081954

**Published:** 2019-10-09

**Authors:** Lameck Ssemogerere, Cornelius Sendagire, Ceaser Mbabazi, Yvonne Namungoma, Anna Noland Oketayot, Judith Namuyonga, Cephas Mijumbi, Ritah Nkwine, Moses Othin, Michael Oketcho, John Paul Magala, Peter Lwabi, Arthur Kwizera, Martin W. Dünser, Christine Florence Najjuka

**Affiliations:** ^1^Department of Anaesthesia and Critical Care, Makerere University College of Health Science, Kampala, Uganda; ^2^Cardiac Critical Care Research Group-Uganda (CCCRG-Ug), Kampala, Uganda; ^3^Uganda Heart Institute, Kampala, Uganda; ^4^Department of Anaesthesiology and Intensive Care Medicine, Kepler University Hospital and Johannes Kepler University, Linz, Austria; ^5^Department of Medical Microbiology, Makerere University College of Health Science, Kampala, Uganda

## Abstract

**Background:**

Hands of healthcare workers (HCWs) are vehicles for pathogens responsible for healthcare-associated infections (HAIs). Following the identification of Gram-negative organisms (GNOs) in all cases of HAIs in the cardiac intensive care unit (ICU), we sought to determine the burden of hand colonization with GNOs among healthcare workers who access the cardiac ICU.

**Methods:**

We retrospectively reviewed results from surveillance cultures of fingertip imprints of HCWs who access the cardiac ICU at the Uganda Heart Institute. We collected data on staff category, isolates, and susceptibility to antibiotics. We analyzed the data using Microsoft Excel, and the results are summarized in proportions and percentages and presented in charts and tables.

**Results:**

Fifty-six healthcare workers participated in the surveillance. 21 were ICU clinicians, 21 non-ICU clinicians, and 14 nonclinicians. GNOs were cultured in 19 (33.9%) HCWs, in which 8/19 (42.1%) were non-ICU clinicians, 6/19 (31.2%) ICU clinicians, and 5/19 (26.3%) nonclinicians. 32 isolates were identified, of which 47%, 28%, and 25% were cultured from non-ICU clinicians, nonclinicians, and ICU clinicians, respectively. Predominant isolates were *Acinetobacter* (34%), *Citrobacter* (21.9%), and *Pseudomonas* (21.9%). Antimicrobial resistance ranged from 4% to 90%. 9/28 (32.1%) isolates, predominantly *Acinetobacter* species (spp), were carbapenem resistant. 8/28 (28.6%) isolates, predominantly *Citrobacter* spp, were multidrug resistant. Resistance to ciprofloxacin and cefepime was low at 3.6% and 4.4%, respectively.

**Conclusion:**

Gram-negative organisms, predominantly *Acinetobacter*, *Citrobacter*, and *Pseudomonas* spp, were prevalent on the hands of HCWs who access the cardiac ICU irrespective of the staff category. Antimicrobial resistance was high, with multidrug resistance and carbapenem resistance common among *Citrobacter* spp and *Acinetobacter* spp, respectively. Resistance to cefepime and ciprofloxacin was low.

## 1. Background

Healthcare-associated infections (HAIs) remain a major threat to healthcare worldwide. HAIs in the intensive care unit are associated with very poor ICU and hospital outcomes [1–4]. Furthermore, HAIs in the ICU have worse outcomes compared to non-ICU-acquired infections [5]. Transmission of these infections to patients is mainly via the hands of healthcare workers (HCWs). The colonized HCWs and ICU/hospital environment serve as reservoirs and the main risk factor for HAIs. [6–10].

Pathogenic Gram-negative organisms (GNOs) are commonly identified in patients with HAIs. They colonize patients, HCWs, and hospital environments during routine patient care and are common residents in the ICUs and other hospital wards [4, 6, 8, 11, 12]. The most prevalent pathogenic GNOs in health care worldwide are *Acinetobacter*, *Pseudomonas*, and *Citrobacter* spp [13–17]. The similarity of pathogens causing HAIs to those colonizing HCWs' hands and hospital environment has been demonstrated [9]. In one study, 21% of the infections were caused by GNOs found on personnel hands, and the predominant hand colonizers were found to be *Acinetobacter* (45%) and *Klebsiella* (39%) [9, 18].

Frequently, pathogenic GNOs colonizing staff hands and the hospital environment are multidrug resistant. Their potential to produce extended spectrum beta-lactamase (ESBL) and carbapenemase enzymes contributes to antimicrobial resistance and multidrug resistance (MDR) [17, 19–21]. At Mulago Hospital in Uganda, a study revealed carbapenem-resistant GNOs were present [22]. The multidrug resistance makes GNOs very difficult to treat, leading to higher costs of healthcare and poor ICU and hospital outcomes [4, 13, 23, 24]. Noteworthy, these organisms can persist in the colonized environment and cause infection outbreaks [16, 25, 26]. With knowledge of such characteristics, deliberate efforts are needed to prevent colonization of the hospital environment and HCWs. To prevent transmission of pathogenic GNOs and other bacteria from patients to the hospital environment and vice versa, strict infection prevention and control (IPC) practices including hand hygiene are mandated. Proper hand hygiene reduces hand colonization with infection and is a superior practice in the prevention of infection transmission but requires HCWs' compliance with optimal practices [8, 27].

In Uganda, we lack evidence on colonization of HCWs' hands with GNOs and on IPC practices in the ICUs and other wards, yet MDR GNOs have been identified consistently from patients with HAIs admitted to these units [17, 22, 28]. The purpose of the study therefore was to determine the prevalence and antimicrobial susceptibility patterns of GNOs colonizing the hands of HCWs who have access to the cardiac ICU at the Uganda Heart Institute (UHI).

## 2. Methods

### 2.1. Study Design

This was a retrospective cross-sectional study conducted at UHI following the approval from UHI. We reviewed data generated during the surveillance activity by UHI in conjunction with the Department of Medical Microbiology, Makerere University College of Health Sciences (MakCHS). The surveillance lasted a week. Fingertip imprints were collected from HCWs who have access to the ICU. Samples were taken for culture and antibiotic susceptibility testing. The surveillance was specific for GNOs which were the only organisms identified from septic patients in the ICU between 2016 and 2018.

### 2.2. Study Setting

Uganda Heart Institute is a national referral center for cardiovascular services and the only facility in the country with the capacity to conduct cardiac surgery, both open and closed. UHI has one closed-type cardiac ICU with four fully functional beds. The unit admits up to 100 postoperative and approximately 200 nonsurgical cardiac patients per year. The ICU is run full time by a dedicated team of cardiac critical care specialists and operates with a 1 : 1 patient to nurse ratio at all times.

Non-ICU staff (both clinical and nonclinical) frequently have access to the ICU to provide specific services. They include cardiologists, surgeons, radiographers, theatre nurses, physiotherapists, biomedical technicians, catheterization laboratory nurses, resident doctors, and support staff. To reduce on the risk of postoperative infection, all patients undergoing surgery at UHI receive the World Health Organization (WHO) recommended prophylactic antibiotics before skin incision. Postoperatively, antibiotics are continued until all invasive catheters are removed. On average, patients receive prophylactic antibiotics for two to three days postoperatively. Cefuroxime is used at UHI for prophylaxis. Occasionally, other antibiotics like amikacin or gentamicin are added when sensitive GNOs are suspected.

### 2.3. Sample Collection and Processing

Sample collection and processing was led by the Department of Medical Microbiology at MakCHS. The department participated in proficiency testing by the American College of Pathologists (CAP NO. 732255-93-01).

Fingertip impressions were rolled on MacConkey media with and without cefotaxime 2 *μ*g/ml and streaked for pure colonies. Samples were delivered to the lab within two hours of collection. The plates were incubated for 18–24 h at 35–37°C. Resulting colonies were subjected to further conventional biochemical tests for definitive identification. Antibiotic susceptibility testing was performed using the Kirby–Bauer disc diffusion method according to the Clinical and Laboratory Standards Institute (CLSI) standards 2016 [29]. Carbapenemase production testing was carried out using the modified carbapenem inactivation method [30]. PCR detection for beta-lactamase genes (bla genes) was not performed. Results were available electronically and on hard paper.

### 2.4. Data Collection and Analysis

We reviewed all records of the results from surveillance cultures of fingertip imprints done on HCWs who participated in the surveillance. We did not exclude any record of the results. We collected data on HCWs' designations, isolates, organism species, antibiotics, and susceptibility to antibiotics. We categorized HCWs as ICU clinicians (ICU-C), non-ICU clinicians (nICU-C), and nonclinicians (NC). In this study, clinicians refer to physicians and nurses while nonclinicians refer to all other staff. We grouped isolates under GNO species identified. For susceptibility to antibiotics, we assumed all intermediate and resistant isolates were nonsusceptible; herein, referred to as “resistant.” Resistance to three or more classes of antibiotics was defined as MDR. We analyzed data using Microsoft Excel, and the results are summarized in proportions and percentages and presented in tables and charts.

## 3. Results

### 3.1. Staff Distribution

**Figure 1:**
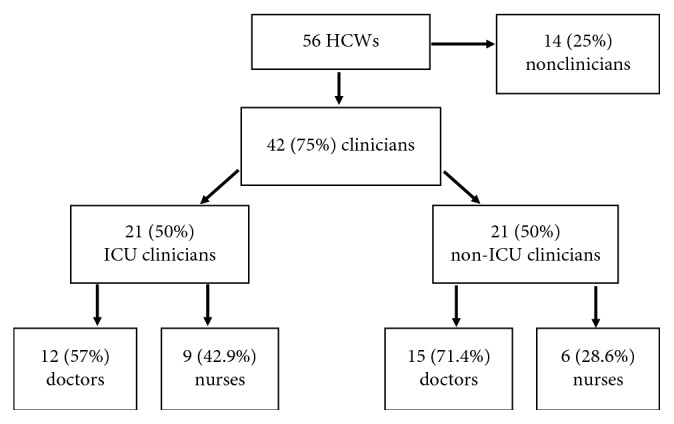
Flow chart showing distribution of HCWs.

Fifty-six healthcare workers participated in the surveillance. 42/56 (75%) were clinicians and 14/56 (25%) were nonclinicians (NC). 50% (21/42) of the clinicians were ICU clinicians (ICU-C). Doctors were more than 50% in both ICU-C and nICU-C categories (Figure 1).

### 3.2. Culture Outcomes

**Figure 2:**
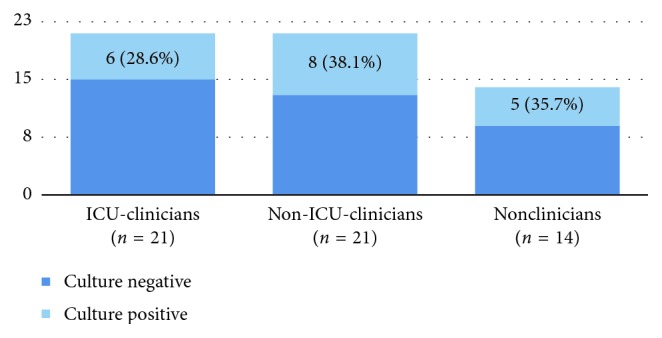
Proportion of culture-positive staff per staff category.

Nineteen (33.9%) HCWs had positive cultures for GNOs. 5/19 (26.3%) were NC, 6/19 (31.6%) were ICU-C, and 8/19 (42.1%) were nICU-C. Non-ICU clinicians had the highest proportion of staff with positive cultures (Figure 2).

A total of 32 isolates and six GNO species were identified from 19 healthcare workers. 15/32 (46.9%) of the isolates were identified form non-ICU clinicians (Table 1).

### 3.3. Carbapenemase and ESBL Production

**Figure 3:**
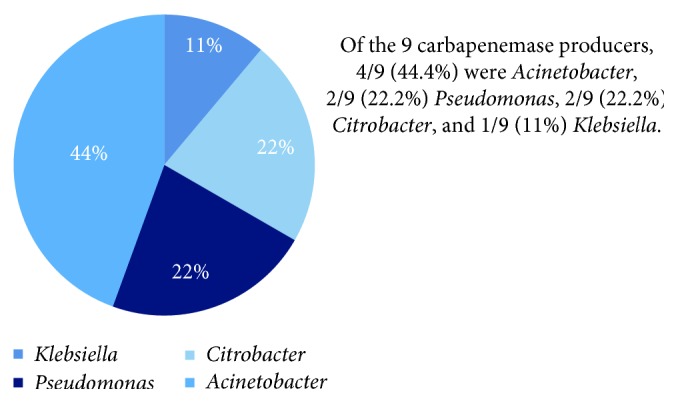
Distribution of carbapenemase-producing GNO isolates per species.

Twenty-eight (28) isolates were checked for susceptibility to antibiotics, and for carbapenemase, and ESBL production. 9 (32.1%) isolates, predominantly *Acinetobacter* spp, were carbapenemase producers (Figure 3). Of the nine, 5 were from ICU clinicians, 3 from non-ICU clinicians, and 1 from nonclinician. Three isolates (*Acinetobacter*, *Klebsiella*, and *Enterobacter* spp) were ESBL producers.

### 3.4. Antimicrobial Susceptibility

Resistance to antibiotics ranged from 4% to 90%. The lowest resistance was to ciprofloxacin and cefepime, at 3.6% and 4.4%, respectively (Table 2).

We found multidrug resistance in 8/28 (28.6%) isolates. *Citrobacter* spp were the most prevalent MDR isolates (4/8) (Table 3). Of the eight MDR isolates, four (50%) and three (37.5%) were identified from ICU clinicians and non-ICU clinicians, respectively.

*Acinetobacter* spp and *Citrobacter* spp were the predominant carbapenem and MDR isolates, respectively. The majority of *Pseudomonas* isolates were pan-sensitive. Noteworthy, only one *Acinetobacter* isolate was resistant to cotrimoxazole. In general, resistance to cefepime, ciprofloxacin, gentamicin, and cotrimoxazole was low (Table 4).

## 4. Discussion

Gram-negative organisms were prevalent on hands of HCWs who have access to the ICU at UHI. *Acinetobacter*, *Citrobacter*, and *Pseudomonas* species were the most common GNOs identified. Multidrug and carbapenem resistance were common, predominantly MDR-*Citrobacter* spp and carbapenem-resistant *Acinetobacter* spp. Other MDR isolates identified were *Klebsiella*, *Enterobacter*, and *Serratia* spp. The pathogens and their characteristics in this study are similar to those previously identified in studies conducted on patients at Mulago National Referral Hospital, Uganda [17, 22, 28].

This study offers the first research evidence on colonization of HCWs' hands with GNOs in Uganda. With all the available evidence on the role of HCWs' hands in the transmission of HAIs [31–33], we hypothesized that septic patients in the cardiac ICU could have been infected via colonized hands of HCWs. It is not uncommon for HCWs' hands to get colonized during routine patient care and handling of contaminated surfaces in the ICU and other hospital environments [7, 9, 26, 32–34].

In this study, a third of HCWs who participated in the surveillance had GNOs cultured from their hands. Even though most isolates where identified from non-ICU clinicians, the majority of MDR and carbapenem-resistant isolates were identified from ICU clinicians. Nonclinicians who included cleaners, biomedical engineers, physiotherapists, and others were the fewest participants and majority of them were colonized. These colonization patterns have been reported in a few other studies [11, 32, 33]. Nonclinicians with the exception of physiotherapists rarely have direct contact with patients but routinely make contact with surfaces in the ICU and hospital environment. This puts them at equal risk of contamination and colonization thereafter becoming reservoirs and vehicles for pathogens [9, 26, 32].

Variations in standards of IPC practices and their implementation in the different units at the institute may partly explain why more non-ICU clinicians compared to ICU clinicians could be colonized with GNOs. The strict IPC practices in the ICU play a major role in the control of hand colonization and transmission of infection. However, to further reduce the risk of colonization of HCWs' hands, standardized IPC guidelines must be implemented and adhered to in all sections of the hospital. Non-ICU clinicians and nonclinicians move frequently between the ICU and other areas in the hospital. These inevitable movements between places in a setting without adequate and accessible proper hand hygiene facilities promote poor hand hygiene and hand colonization with pathogenic microbes. [8, 18, 35–37].

The movement of HCWs between wards and hospitals can be associated with the colonization of the different wards and hospitals by the same pathogens [12]. This may explain why HCWs from the different staff categories were colonized by similar GNOs. Nonclinicians just like the clinicians are continuously exposed to colonized hospital environments, hence the resemblance in colonization patterns [6, 10, 26, 37].

In general, ICU clinicians follow strict IPC guidelines including use of strong disinfectants, multiple hand antiseptics, and high-end antibiotics. Consequently, ICU clinicians are usually colonized by MDR pathogens. These organisms persist in the ICU environment and are difficult to eliminate, making ICUs permanent reservoirs [12, 18]. In this study, most of the MDR and carbapenem-resistant organisms were identified from the hands of ICU clinicians. These MDR organisms were similar to those found in previous studies on septic patients at Mulago Hospital in Uganda. This observation reflects the possibility of an already colonized hospital environment specifically ICU [17, 22, 28]. This implies that the hospital environment and clinicians are potential reservoirs for MDR GNOs. The potential for contamination of HCWs with organisms from a colonized work environment has been confirmed in some studies [9, 26, 33, 34].

*Acinetobacter* was the most prevalent GNO isolate cultured from the hands of HCWs (34.4%). This has been a consistent finding in many studies on GNO in the ICU and in patients with HAIs [4, 17, 18, 33, 38, 39]. *Acinetobacter* species continue to emerge as the most frequent high-prevalent GNO identified in HAIs in ICUs, on wards, and on HCWs and this has been demonstrated repeatedly [17, 22, 33, 40]. *Pseudomonas* and *Citrobacter* had equal prevalence (21.9%) and were the second commonest GNO isolate in this study. Unlike *Pseudomonas*, *Citrobacter* is relatively new as a cause of HAIs. It is currently one of the commonly isolated GNO in HAIs and a major threat to health care due to its ability to become MDR. [15, 33, 41–43].

*Acinetobacter* was the most prevalent carbapenem-resistant isolate (4/9) followed by *Pseudomonas* (2/9) and *Citrobacter* (2/9). These characteristics have been demonstrated before in other studies [20, 21, 44]. A majority of *Acinetobacter* isolates were resistant to piperacillin (54.4%) and to meropenem (36.4%), while *Citrobacter* isolates had a 28.6% resistance to meropenem. The single *Klebsiella* isolate and one *Acinetobacter* isolate exhibited extensive drug resistance. These resistance patterns are similar to those in a study done at Mulago Hospital, Uganda, on antimicrobial resistance in hospitalized surgical patients and another on nosocomial bacterial infections and their susceptibility patterns among ICU patients in Uganda [17, 28]. These studies demonstrated MDR characteristics amongst GNOs similar to those shown in this study. The findings of this study identify the emerging threat of MDR GNOs and carbapenem-resistant organisms in the ICU at UHI with a predominance of *Acinetobacter* which is known to persist in environments. This threat is a worldwide concern as described in multiple studies [45–48].

To prevent colonization of HCWs with pathogenic GNOs and other bacteria as well as transmission to patients and hospital environment, strict IPC must be implemented. These include proper hand hygiene as defined by the World Health Organization, surveillance for HAIs, antibiotic stewardship, and training of HCWs on infection and IPCs. Proper hand hygiene specifically hand washing reduces hand colonization with infections and is a superior practice in the prevention of infection transmission but requires HCWs' compliance with optimal practices [8, 27].

At UHI, IPC was only emphasized and partially implemented in the ICU and operating theatre. There were no standardized guidelines on IPC for the hospital. Additionally, the facilities for hand hygiene such as hand washing basins and antimicrobial dispensers were few. This could have contributed to the emergency of sepsis caused by multidrug and carbapenem-resistant GNO observed in the cardiac ICU. There was no evidence of adherence to appropriate hand hygiene practices across the institute. Without proper hospital IPC guidelines, the risk of HAIs and colonization of both HCWs and hospital environment is magnified. The objective of the surveillance therefore was to identify the presence of carbapenem-resistant Gram-negative bacteria in the animate and inanimate environment as a likely source of what was observed in patients. The findings as discussed in this study were the basis for the establishment and funding of the IPC committee at UHI. The committee audits IPC practices, ensures training of staff on IPC and availability of appropriate hand hygiene facilities including antimicrobial dispensers, and monitors the implementation of standardized IPC guidelines. These interventions are expected to yield favorable outcomes in regard to IPC at UHI.

## 5. Limitations

A few of HCWs who access ICU were not present at the time of collecting fingerprint samples. This obviously affects the proportions and percentages reported leading to either exaggeration or underreporting of results. The retrospective study design may have introduced selection and information bias. The findings of this study may not be adequate to influence practice in other settings.

## 6. Conclusion

GNOs were prevalent on the hands of HCWs who accessed the cardiac ICU in Uganda. *Acinetobacter*, *Citrobacter*, and *Pseudomonas* species were the most common GNOs identified. Both MDR and carbapenemase resistance were common. Half of the MDR GNOs were *Citrobacter*, while half of the carbapenem-resistant GNOs were *Acinetobacter*. Resistance to cefepime and ciprofloxacin was low.

## Figures and Tables

**Table 1:** Frequency and distribution of GNO isolates among categories of HCWs (*n* = 32).

Organisms	Frequency (%)	ICU clinicians	Non-ICU clinicians	Nonclinicians
*Acinetobacter*	11 (34.4)	3	3	5
*Citrobacter*	7 (21.9)	1	4	2
*Pseudomonas*	7 (21.9)	0	7	0
*Klebsiella*	1 (3.1)	1	0	0
*Serratia*	1 (3.1)	1	0	0
*Bacillus*	4 (12.5)	2	1	1
*Enterobacter*	1 (3.1)	0	0	1
Total isolates	**32**	**8**	**15**	**9**

**Table 2:** Antimicrobial resistance of isolates.

Antibiotics	Isolates exposed	Resistant isolates	Proportion (%)
Ciprofloxacin	28	1	1/28 (3.6)
Cefepime	23	1	1/23 (4.4)
Tazobactam	28	3	3/28 (10.7)
Gentamicin	28	4	4/28 (14.3)
Cotrimoxazole	21	3	3/21 (14.3)
Ceftazidime	28	6	6/28 (21.4)
Ceftriaxone	10	3	3/10 (30.0)
Chloramphenicol	10	3	3/10 (30.0)
Meropenem	28	9	9/28 (32.1)
Piperacillin	16	6	6/16 (37.5)
Amoxycillin-clavulanate	10	4	4/10 (40.0)
Cefuroxime	10	5	5/10 (50.0)
Cefotaxime	7	5	5/7 (71.4)
Ampicillin	10	9	9/10 (90.0)

**Table 3:** Distribution of MDR isolates by GNO.

Organisms	Frequency of isolates	Frequency of MDR
*Acinetobacter*	11	1
*Citrobacter*	7	4
*Pseudomonas*	7	0
*Klebsiella*	1	1
*Serratia*	1	1
*Enterobacter*	1	1
Total isolates	**28**	**8 (28.6%)**

**Table 4:** GNO species specific antimicrobial resistance.

Antibiotics	ACIN (*N* = 11)	CIT (*N* = 7)	PSEUD (*N* = 7)	KLEB (*N* = 1)	SER (*N* = 1)	ENT (*N* = 1)
Ampicillin	x	6	x	1	1	1
Amoxycillin-clavulanate	x	1	x	1	1	1
Piperacillin	6	x	0	x	x	x
Tazobactam	1	2	0	0	0	0
Cefuroxime	x	3	x	0	1	1
Ceftriaxone	x	1	x	1	0	1
Cefotaxime	x	3	x	1	1	x
Ceftazidime	2	2	0	1	0	1
Cefepime	1	0	0	x	x	x
Meropenem	4	2	2	1	0	0
Ciprofloxacin	1	0	0	0	0	0
Gentamicin	1	1	0	1	0	1
Cotrimoxazole	1	0	x	1	1	0

ACIN = *Acinetobacter*; CIT = *Citrobacter*; PSE = *Pseudomonas*; KLE = *Klebsiella*; SER = *Serratia*; ENT = *Enterobacter*; x = not tested.

## Data Availability

The data used to support the findings of this study are included within the supplementary information file.

## Supplementary Materials

Supplementary Materials The study describes colonization of healthcare workers' hands with GNOs and the antimicrobial resistance patterns of the identified isolates. We analyzed data collected during surveillance for MDR GNO conducted at the Uganda Heart Institute (UHI). The surveillance was triggered by the emergency of MDR GNO in the cardiac intensive care unit (ICU). Therefore, we focused on healthcare workers who access the cardiac ICU. The prevalence of hand colonization with MDR GNO was high among healthcare workers of all categories who access the cardiac ICU. MDR and carbapenem resistance were common. *Acinetobacter*, *Pseudomonas*, and *Citrobacter* spp were the most prevalent. Resistance to cefepime and ciprofloxacin was low. We emphasize implementation of strict hospital-wide infection prevention and control (IPC) guidelines, hand hygiene inclusive as an immediate and efficient solution as recommended by the WHO.
